# Supplementary material for: CRISPR/Cas9-Mediated Knock-Out of dUTPase in Mice Leads to Early Embryonic Lethality
Source: Biomolecules. 2019 Apr 4;9(4):136. doi: 10.3390/biom9040136 (PMC6523736; doi:10.3390/biom9040136)
Supplement: Supplementary file 1 [file biomolecules-09-00136-s001.pdf]

Supplementary Materials

CRISPR/Cas9-Mediated Knock-Out of dUTPase in Mice Leads to Early Embryonic Lethality

Hajnalka Laura Pálinkás [1,2,3,*], Gergely Attila Rácz [1,3], Zoltán Gál [4], Orsolya Ivett Hoffmann [4], Gergely Tihanyi [1,3], Gergely Róna [3,5,6], Elen Gócza [4,*], László Hiripi [4,*], Beáta G. Vértessy [1,3*]

[1] Institute of Enzymology, RCNS, Hungarian Academy of Sciences, H-1117 Budapest, Hungary; racz.gergely@ttk.mta.hu (G.A.R); tihanyi@stud.uni-heidelberg.de (G.T.)

[2] Doctoral School of Multidisciplinary Medical Science, University of Szeged, H-6720 Szeged, Hungary

[3] Department of Applied Biotechnology and Food Sciences, Budapest University of Technology and Economics, H-1111 Budapest, Hungary ; Gergely.Rona@nyulangone.org (G.R.)

[4] Department of Animal Biotechnology, Agricultural Biotechnology Institute, National Agricultural Research and Innovation Centre, H-2100 Gödöllő, Hungary ; zoltan.gal89@gmail.com (Z.G.); hoffmannorsi@gmail.com (O.I.H.)

[5] Department of Biochemistry and Molecular Pharmacology, New York University School of Medicine, New York, NY10016, United States

[6] Perlmutter Cancer Center, New York University School of Medicine, New York, NY10016, United States

* Correspondence: vertessy@mail.bme.hu (B.G.V); palinkas.hajnalka@ttk.mta.hu (H.L.P.); gocza.elen@abc.naik.hu (E.G.); hiripi1@gmail.com (L.H.)

Received: date; Accepted: date; Published: date

Keywords: dUTPase; CRISPR/Cas9-mediated knock-out; blastocyst outgrowth; embryonic development

(a)

(b)

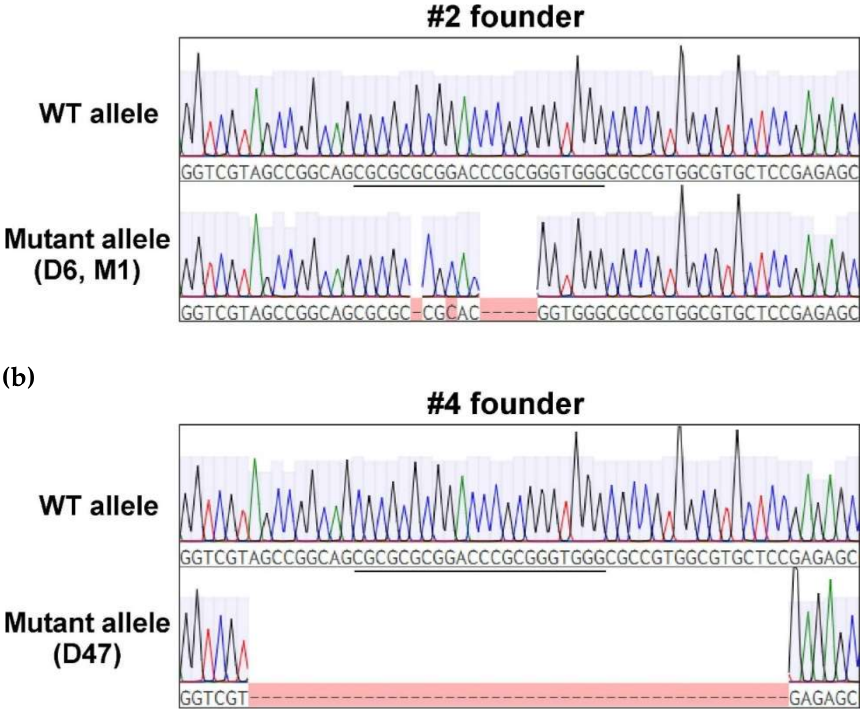

Figure S1. Sequencing analysis of *dut* gene from founder mouse #2 and #4. Sequencing results of *dut* gene from the mouse #2 (**a**) show 6 bp deletion and 1 bp substitution (D6, M1), while from the mouse #4 (**b**) show 47 bp deletion (D47). CRISPR target site including PAM sequence is underlined. CRISPR induced modifications are marked with red highlight.

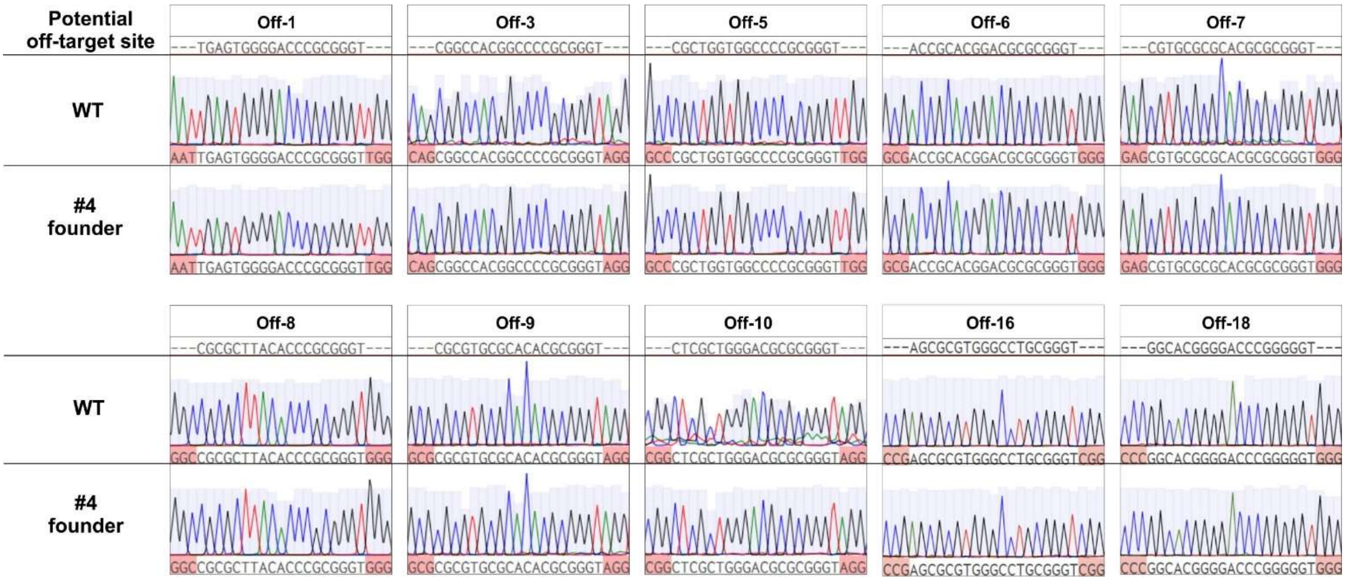

Figure S2. Alignment of the sequencing results from wild type and mouse #4 animals. Sequences are fully identical at these potential off-target sites identified by the CRISPR/Cas9 target online predictor software CCTop.

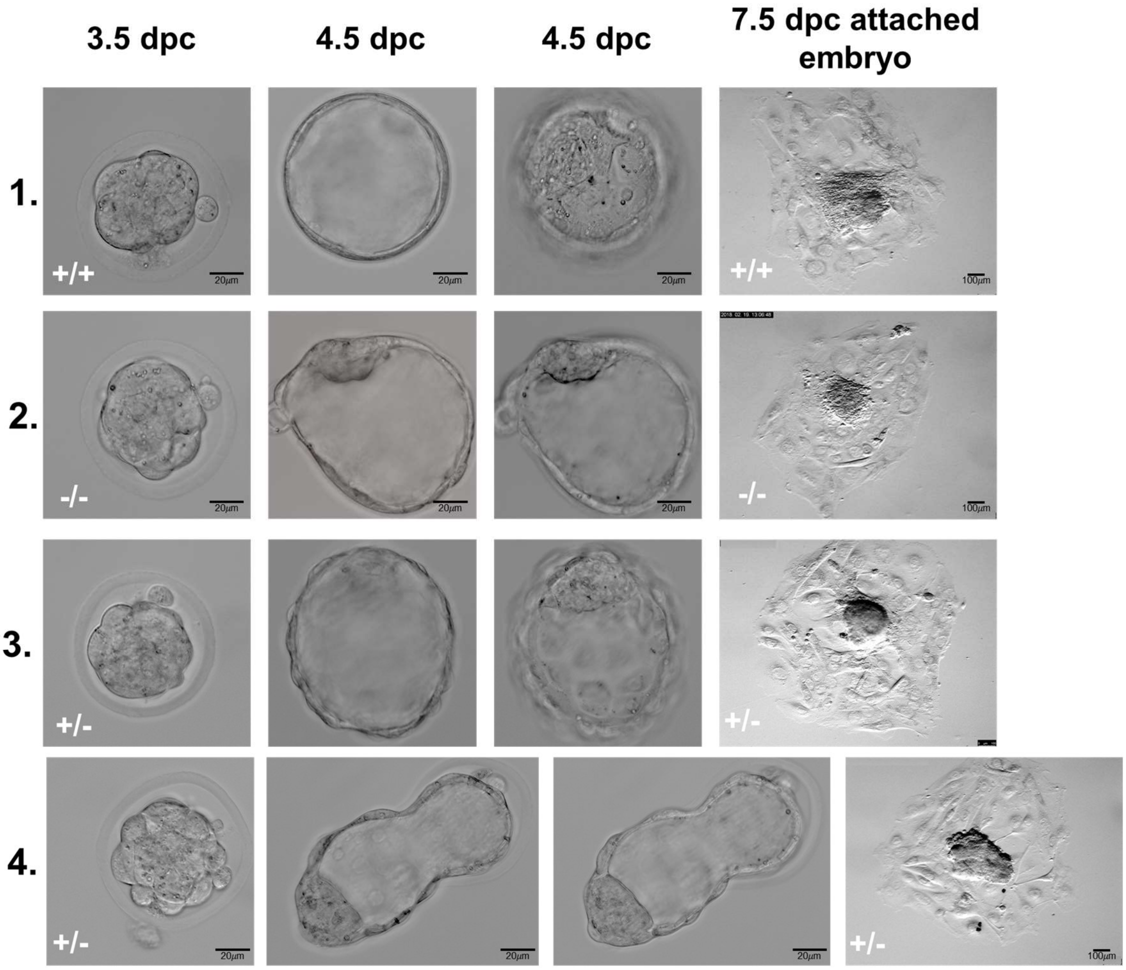
3.5 dpc
4.5 dpc
4.5 dpc
7.5 dpc attached embryo
1.
2.
3.
4.
+/+
-/-
+/-
+/-
+/+
-/-
+/-
+/-
20μm
100μm

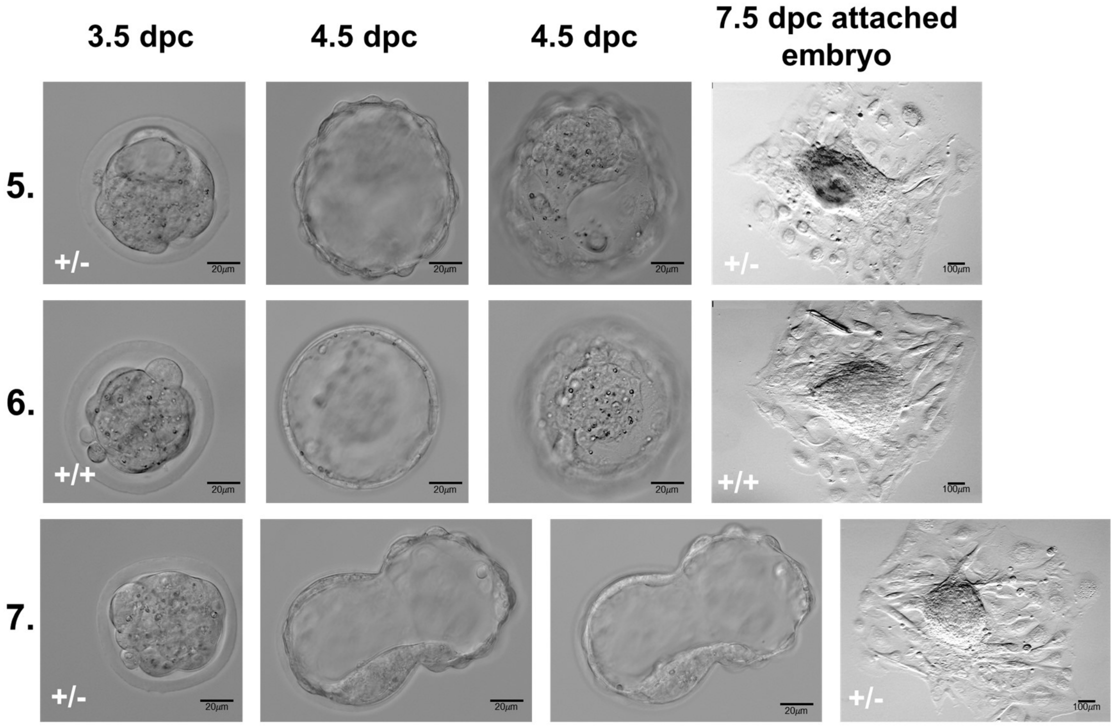
3.5 dpc
4.5 dpc
4.5 dpc
7.5 dpc attached embryo
5.
6.
7.
+/-
+/+
+/-
+/-
+/+
+/-
20μm
100μm

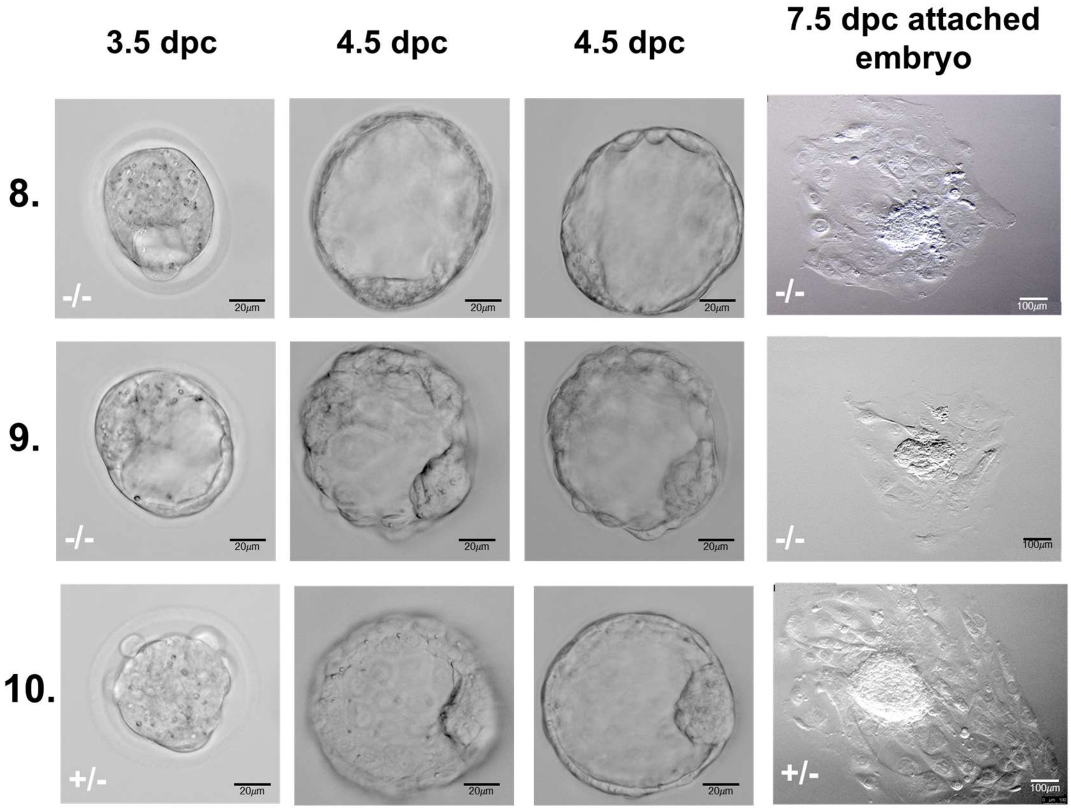

Figure S3. Phase contrast images of embryos obtained by crossing D47 heterozygous mice. Homozygous (*dut* −/−: 2, 8, 9), heterozygous embryos (*dut* +/-: 3, 4, 5, 7, 10), and wild type blastocysts (*dut* +/+: 1, 6) in *in vitro* culture. The first column shows embryos at 3.5 dpc after flushing from oviducts. The second and third columns show the embryos one day later focusing on the trophoblast cells or the inner cell mass (ICM). Scale bar, 20 μm. The last columns present outgrowths after 4 days in culture (7.5 dpc). Scale bar, 100 μm.

(a)

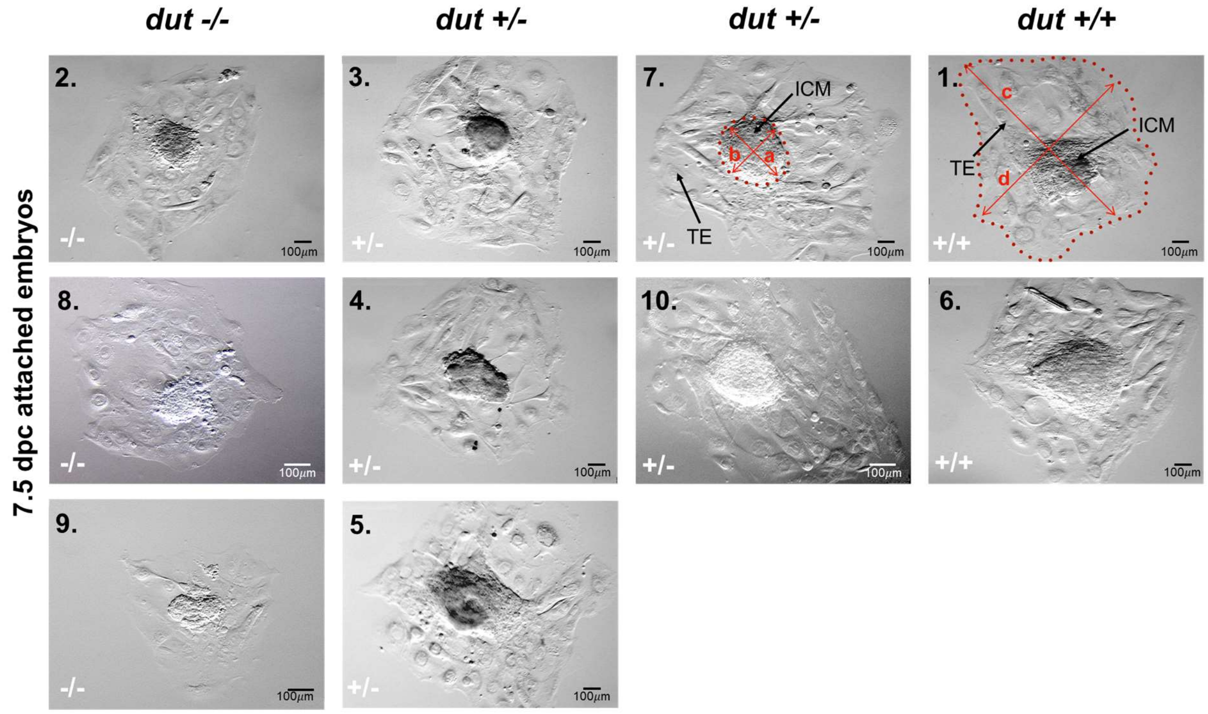

(b)

genotype	number of blastocysts	ICM size (a*b) mm²		TE size (c*d) mm²	
		average	SD(±)	average	SD(±)
dut -/-	3	0.078	0.006	0.964	0.156
dut +/-	5	0.138	0.036	1.425	0.177
dut +/+	2	0.189	0.044	1.314	0.055

Figure S4. Outgrowth assay of preimplantation embryos obtained by intercrossing D47 heterozygous mice. (**a**) The images represent homozygous (*dut* −/−: 2, 8, 9); heterozygous (*dut* +/-: 3, 4, 5, 7, 10); wild type (*dut* +/+: 1, 6) blastocysts attached to the surface of tissue culture dishes and their outgrowth in *in vitro* cultures for 4 days (7.5 dpc). Scale bar, 100 μm. Red arrows (a, b, c, d, respectively) show the parameters used for outgrowth size measurements. (**b**) Table contains the result of the blastocysts outgrowth size measurements: average size of inner cell mass (ICM) and trophectoderm (TE). SD indicates the standard deviation. n = 3 for (*dut* -/-), n = 5 for (*dut* +/-), and n =2 for (*dut* +/+).

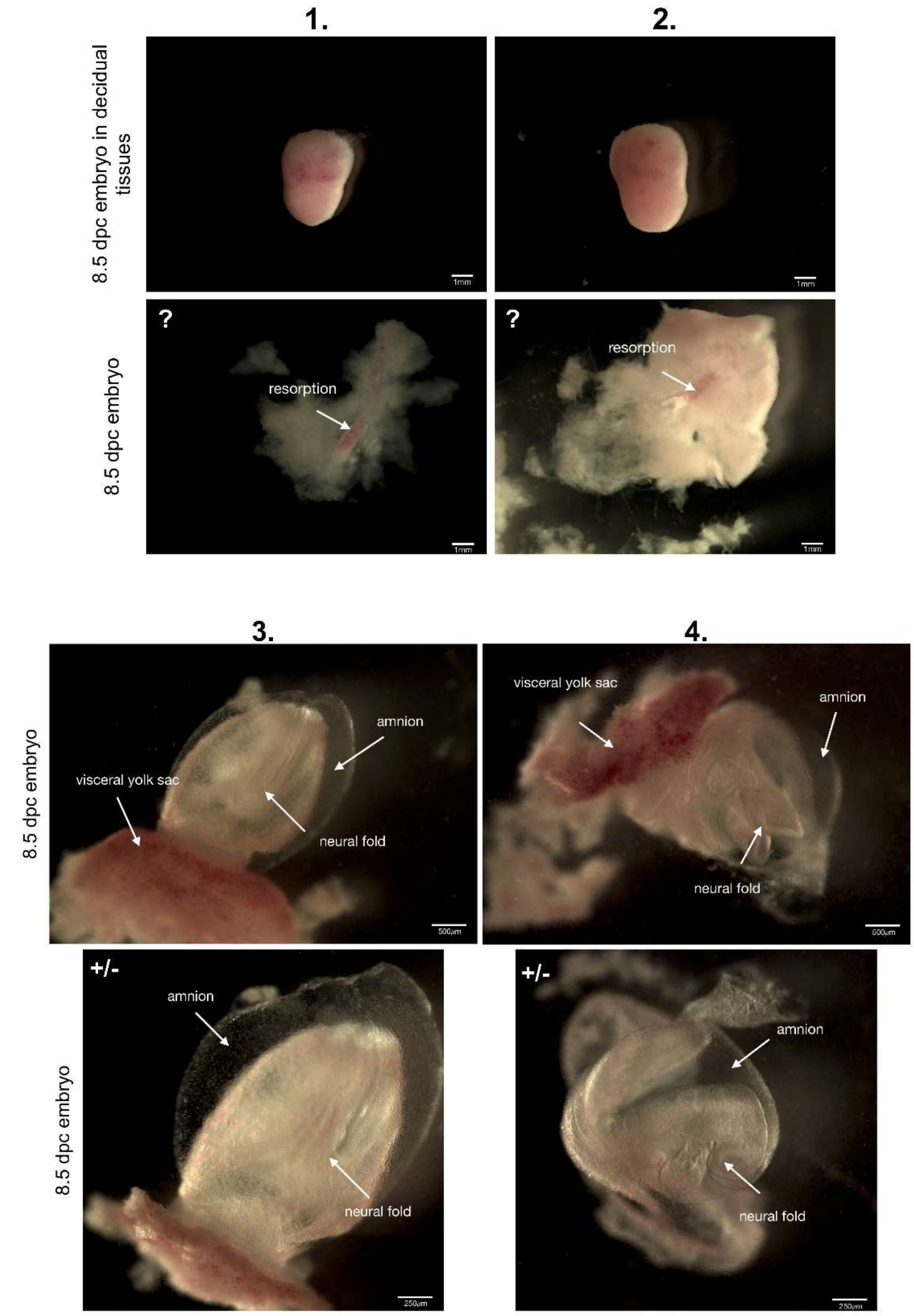
1.
2.
8.5 dpc embryo in decidual tissues
1mm
8.5 dpc embryo
?
resorption
3.
4.
amnion
visceral yolk sac
neural fold
500µm
+/-
250µm

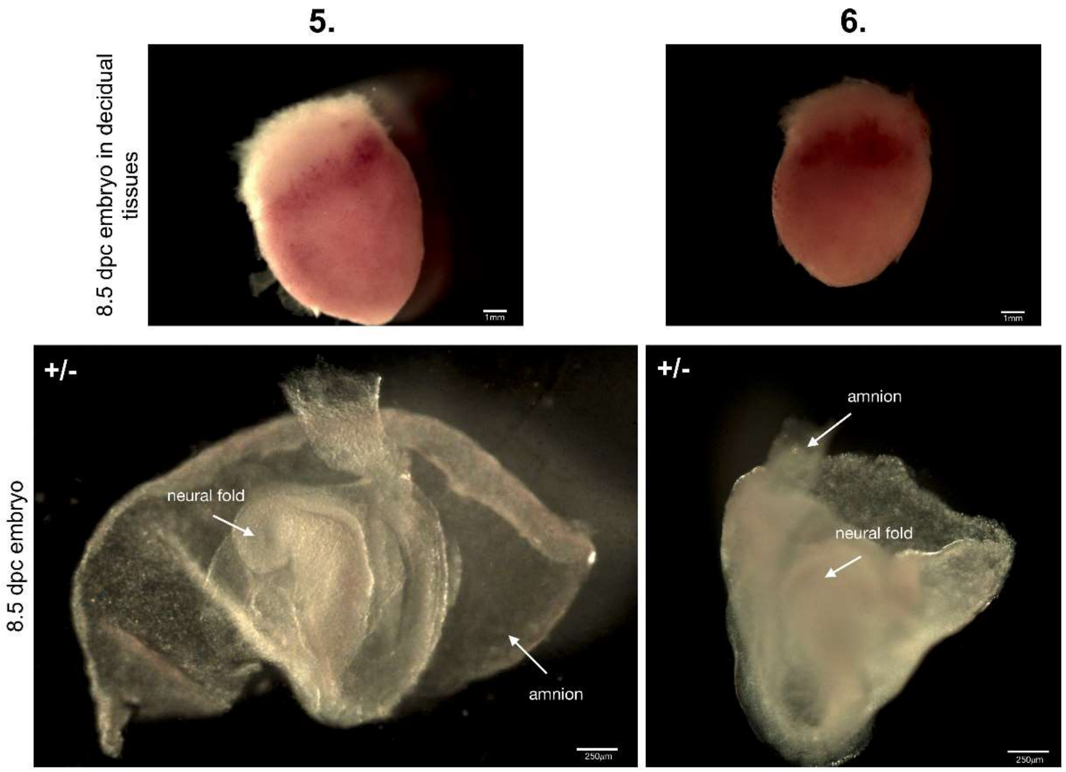
5.
6.
8.5 dpc embryo in decidual tissues
8.5 dpc embryo
+/-
neural fold
amnion
+/-
amnion
neural fold
1mm
250μm

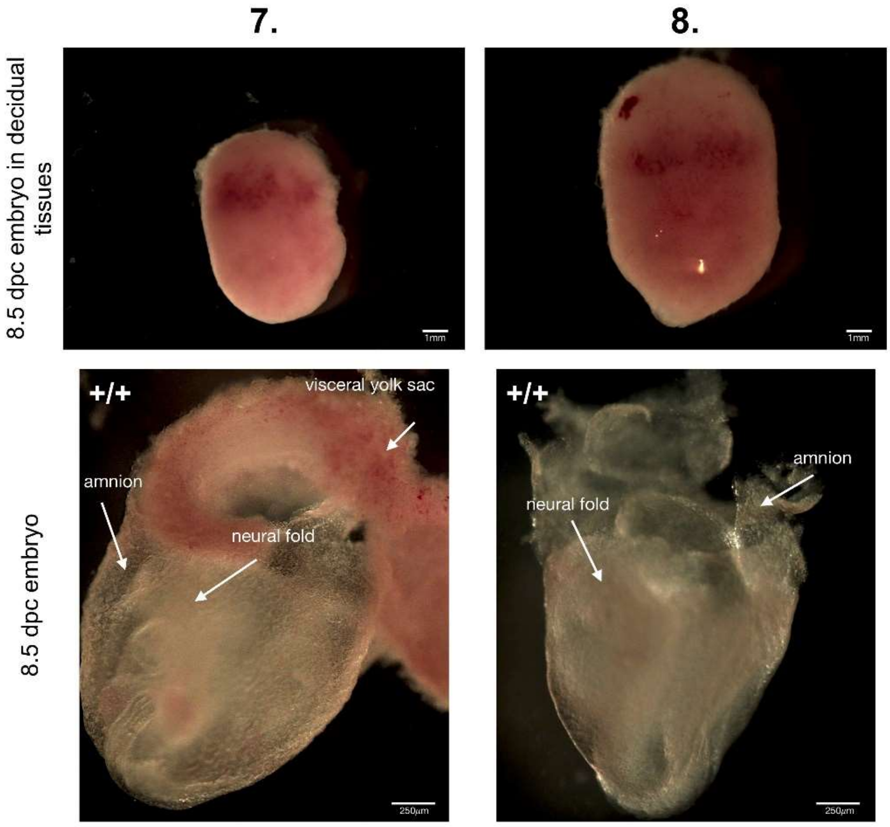
7.
8.
8.5 dpc embryo in decidual tissues
8.5 dpc embryo
+/+
visceral yolk sac
amnion
neural fold
+/+
amnion
neural fold
1mm
250μm

9.

8.5 dpc embryo in decidual tissues

8.5 dpc embryo

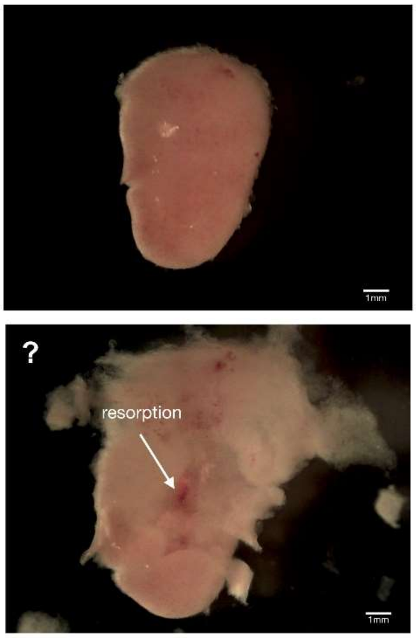

10.

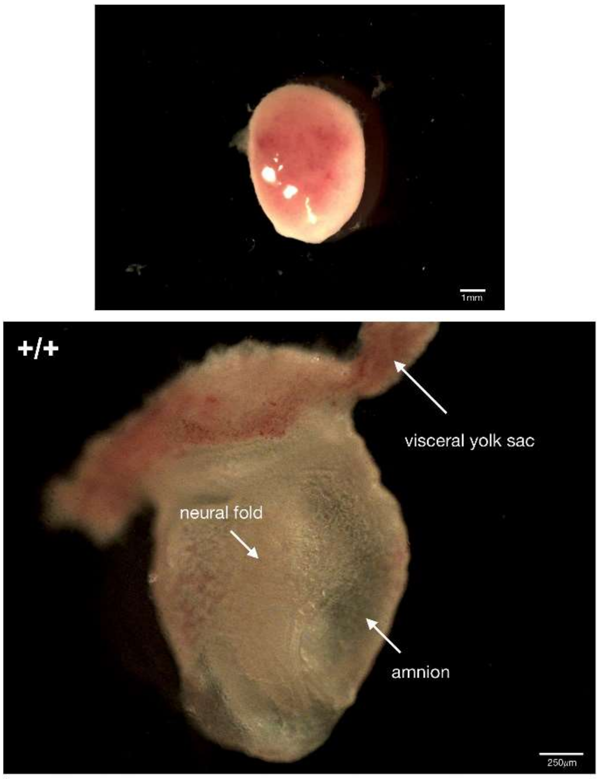

11.

8.5 dpc embryo in decidual tissues

8.5 dpc embryo

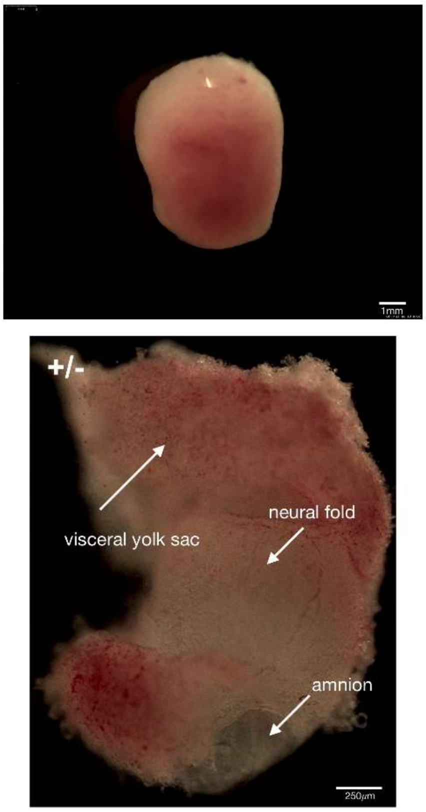

12. 13.

8.5 dpc embryo in decidual tissues

8.5 dpc embryo

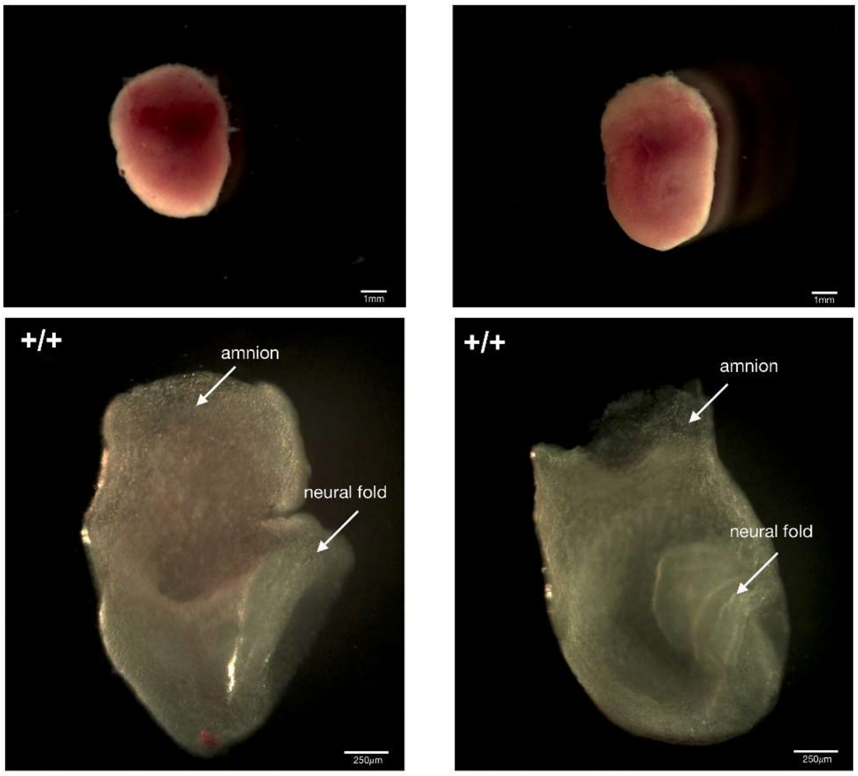

14. 15.

8.5 dpc embryo in decidual tissues

8.5 dpc embryo

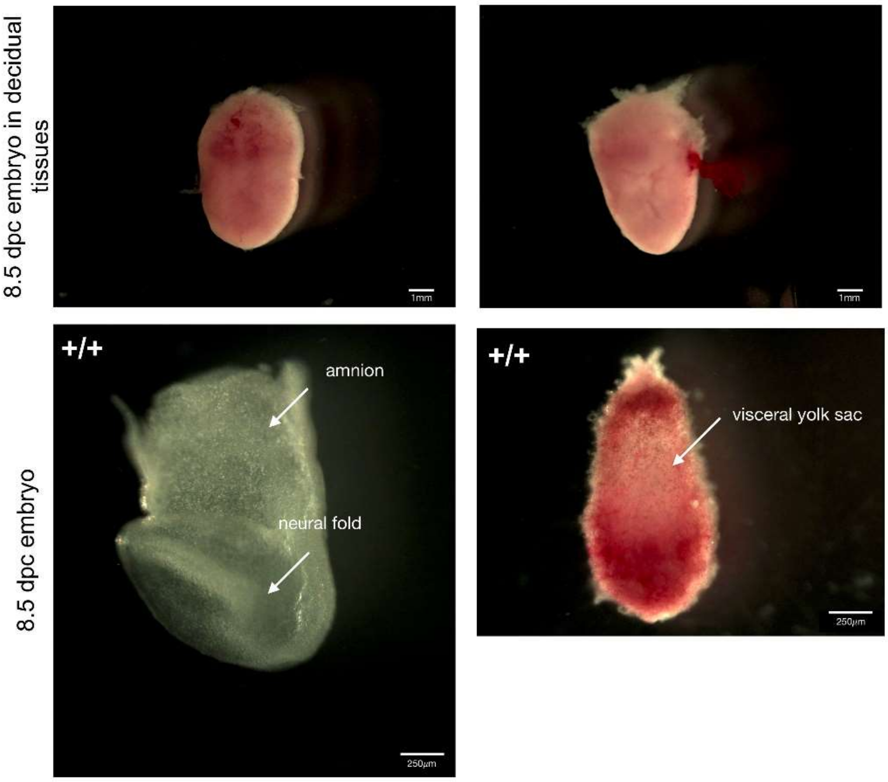

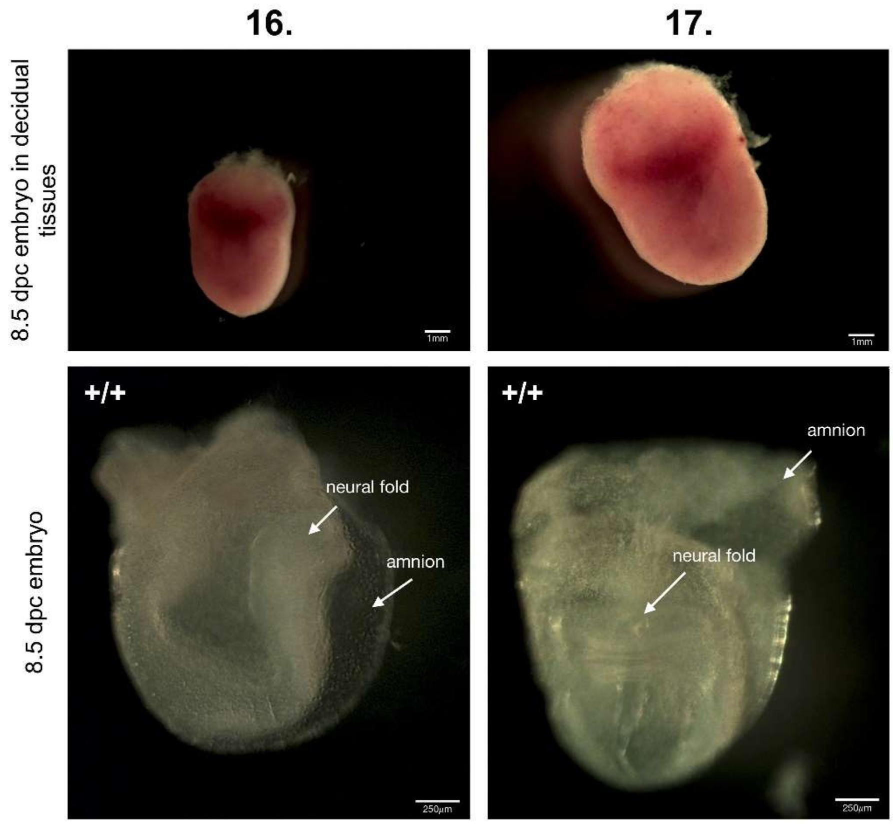
16.
17.
8.5 dpc embryo in decidual tissues
8.5 dpc embryo
+/+
+/+
neural fold
amnion
amnion
neural fold
1mm
1mm
250μm
250μm

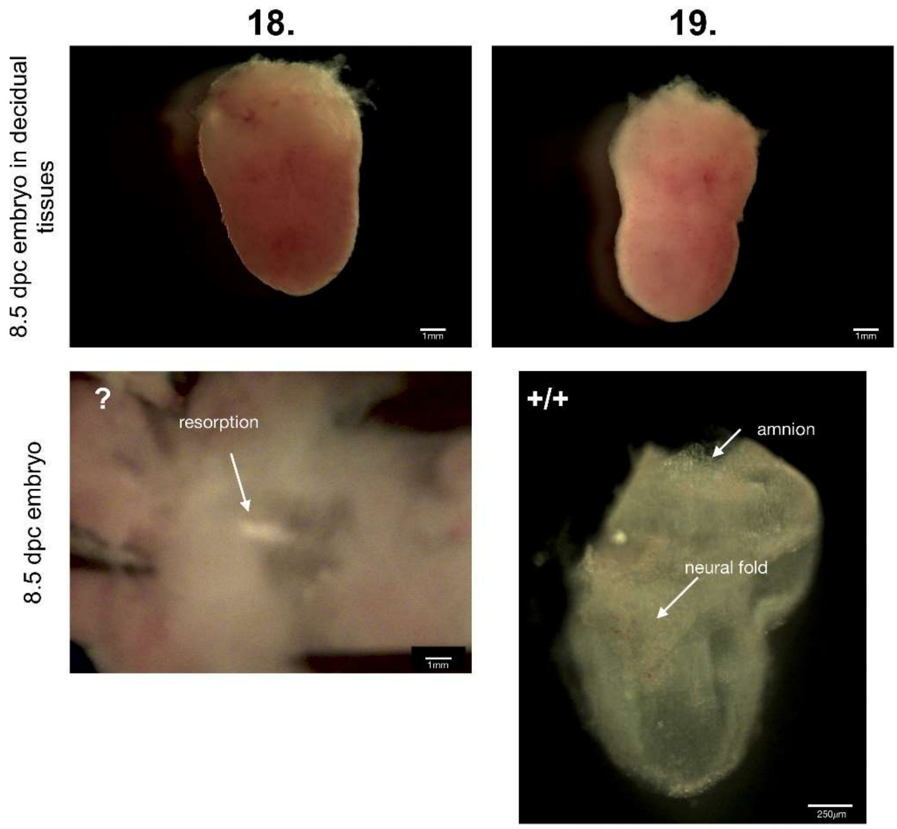
18.
19.
8.5 dpc embryo in decidual tissues
8.5 dpc embryo
?
resorption
+/+
amnion
neural fold
1mm
1mm
1mm
250μm

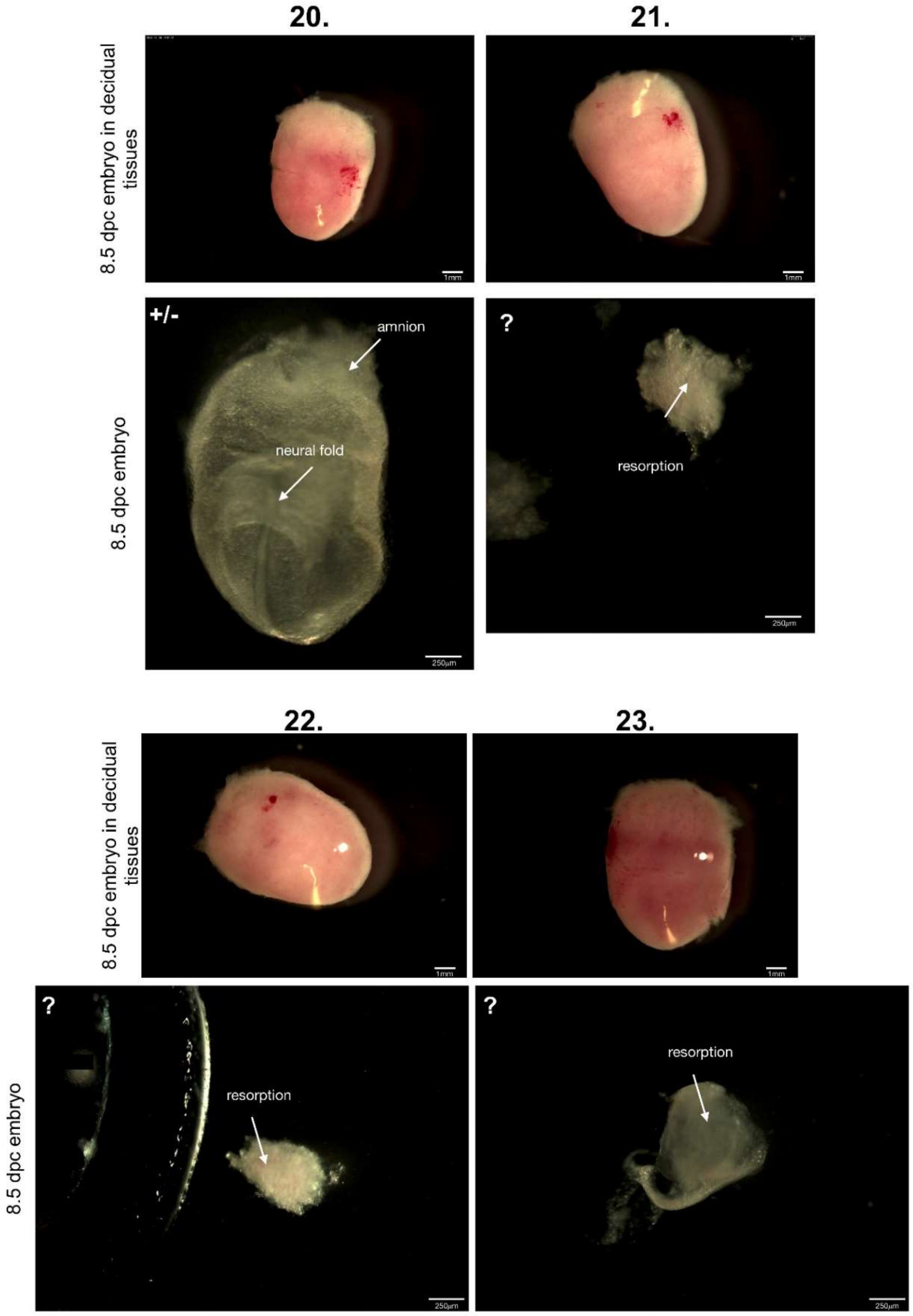

Figure S5. Images of embryos at 8.5 dpc obtained by crossing D47 heterozygous mice. Heterozygous embryos (*dut* +/-: 3, 4, 5, 6, 11, 20) and wild type embryos (*dut* +/+: 7, 8, 10, 12, 13, 14, 15, 16, 17, 19). The resorbed embryos (1, 2, 9, 18, 21, 22, 23) could not be genotyped (indicated with „?"). Upper panels show embryos in the intact decidual tissues. Scale bar, 1 mm. Lower panels present the embryos dissected from decidual tissues. Arrows represent the embryonic regions (neural fold) and extra-embryonic tissues (amnion, yolk sac) of embryos. Scale bar, 250 µm.

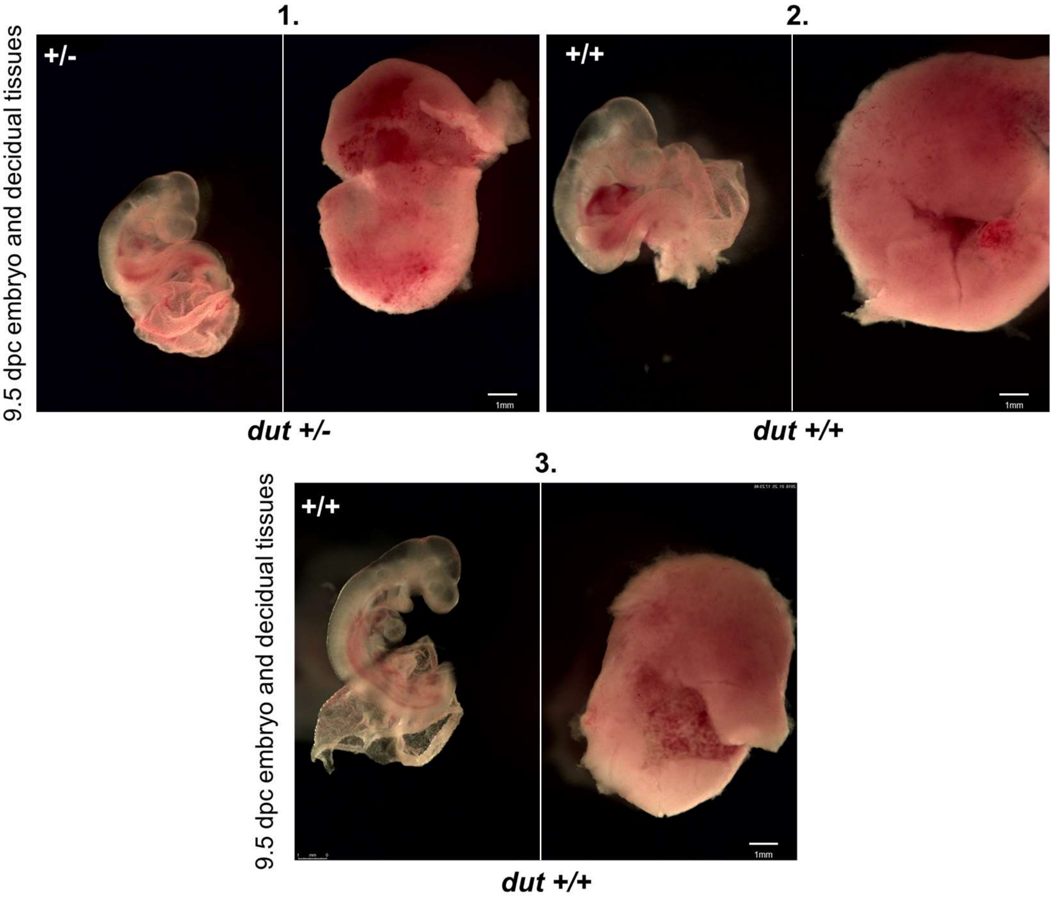

Figure S6. Images of embryos at 9.5 dpc obtained by crossing D47 heterozygous mice. Heterozygous embryos (*dut* +/-: 1) and wild type embryos (*dut* +/+: 2, 3). Right panels show embryos in decidual tissues. Left panels present the embryos dissected from decidual tissues. Scale bar, 1 mm.

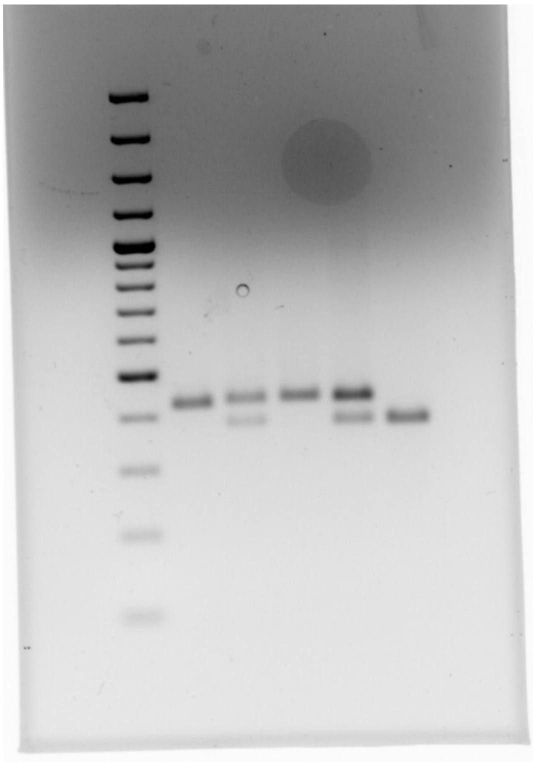

Figure S7. Full-length agarose gel of Figure 2b.

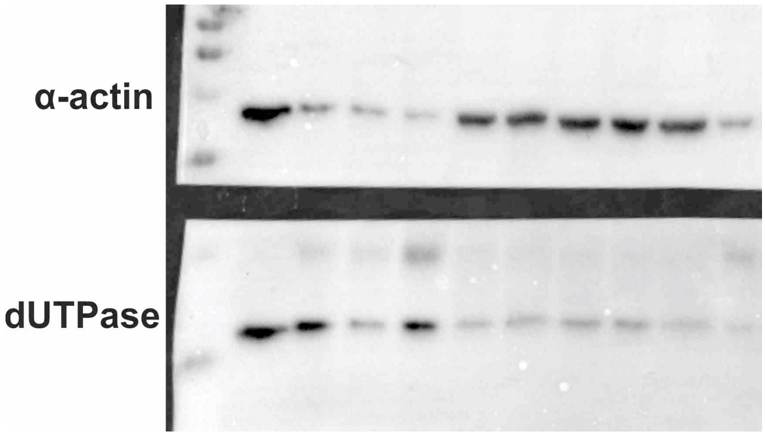

Figure S8. Full-length membrane of Figure 5a.

Table S1. Oligonucleotides used in this study. Restriction sites are underlined.

Sequence (5' - 3')	Name	Experiment
CTGGTCTGAGCAGGTAACGG	Cel-1-F	CEL 1 assay
TAACGTGCACACTGCAGACA	Cel-1-R	
GGTCGGTGCCTCCTCTAG	Dut-gen-F	Genotyping
AATAAGCCTTGCACATCCGG	Dut-gen-R	
GGAGATTTTCGGCGGGTAGG	Dut-nest-F	
TCCGGTCGACCTGGTCTGAGCAGGTAACGG	pBS-F	Cloning
CTAGGAATTCTAACGTGCACACTGCAGACA	pBS-R	
AGCTTTTACTCATTGCTACTCTCT	Off-1-F	Off-target analysis
ATTGTGAAGTGTTGGCTTATGGG	Off-1-R	
CCCCGTTTCACAGAACCGTA	Off-3-F	
CCAAGGACCTAGCCGACATC	Off-3-R	
AACAAGAGCTAGAGGCAGCG	Off-5-F	
CAACAGCAGCTGGGTGTCA	Off-5-R	
GGTGCGTGTTTGCCAGTG	Off-6-F	
GAGAGAAGCAAGCAGATGCG	Off-6-R	
GTCACTATGCAAGAAGTTTCCTTAT	Off-7-F	
CCTACCACGTGCCCTGTAAG	Off-7-R	
CACCTCAGCAGTACCAAACAAAG	Off-8-F	
TTGATGGTAAATTCTCCTCTCTTCA	Off-8-R	
GCAGAATCGAAGGTGGTGGT	Off-9-F	
CAGGGCATAGGATACCTGCT	Off-9-R	
CAAAGGGAATGCCACTTCTCG	Off-10-F	
CGCGCGCACTACTGGAG	Off-10-R	
CGAATGGGATGGTAGCAAGT	Off-16-F	
TTTTGCTGTCAGTCCGTGTT	Off-16-R	
CTTCCTCGCCCTCATCGC	Off-18-F	
GAATGAGCGTTCAGCAGTGG	Off-18-R	
